# Studying primate cognition in a social setting to improve validity and welfare: a literature review highlighting successful approaches

**DOI:** 10.7717/peerj.3649

**Published:** 2017-08-03

**Authors:** Katherine A. Cronin, Sarah L. Jacobson, Kristin E. Bonnie, Lydia M. Hopper

**Affiliations:** 1 Lester E. Fisher Center for the Study and Conservation of Apes, Lincoln Park Zoo, Chicago, IL, United States of America; 2 Department of Psychology, Beloit College, Beloit, WI, United States of America

**Keywords:** Cognition, Primate, Animal welfare, Social, Methodology, Publication trends

## Abstract

**Background:**

Studying animal cognition in a social setting is associated with practical and statistical challenges. However, conducting cognitive research without disturbing species-typical social groups can increase ecological validity, minimize distress, and improve animal welfare. Here, we review the existing literature on cognitive research run with primates in a social setting in order to determine how widespread such testing is and highlight approaches that may guide future research planning.

**Survey Methodology:**

Using Google Scholar to search the terms “primate” “cognition” “experiment” and “social group,” we conducted a systematic literature search covering 16 years (2000–2015 inclusive). We then conducted two supplemental searches within each journal that contained a publication meeting our criteria in the original search, using the terms “primate” and “playback” in one search and the terms “primate” “cognition” and “social group” in the second. The results were used to assess how frequently nonhuman primate cognition has been studied in a social setting (>3 individuals), to gain perspective on the species and topics that have been studied, and to extract successful approaches for social testing.

**Results:**

Our search revealed 248 unique publications in 43 journals encompassing 71 species. The absolute number of publications has increased over years, suggesting viable strategies for studying cognition in social settings. While a wide range of species were studied they were not equally represented, with 19% of the publications reporting data for chimpanzees. Field sites were the most common environment for experiments run in social groups of primates, accounting for more than half of the results. Approaches to mitigating the practical and statistical challenges were identified.

**Discussion:**

This analysis has revealed that the study of primate cognition in a social setting is increasing and taking place across a range of environments. This literature review calls attention to examples that may provide valuable models for researchers wishing to overcome potential practical and statistical challenges to studying cognition in a social setting, ultimately increasing validity and improving the welfare of the primates we study.

## Introduction

The study of animal cognition has a long history that has undergone a general evolution from topics such as self-awareness and whether animals possess human-like language capacities to studies of how animals learn in and navigate social worlds (Beran et al., 2014; Seyfarth & Cheney, 2017). Corresponding with this shift in focus, there has been an increasing interest in studying primate cognition in social environments that reflect the species’ natural history (Cronin & Hopper, 2017). However, implementing cognitive research in a social setting introduces several practical and statistical challenges. Here, we provide a review of publications that have tested primate cognition in a social context in order to provide an overview of the state of social testing in primate cognition research, and present strategies researchers have employed to overcome common challenges.

Testing primate cognition while subjects were isolated from social partners was the norm in the early years of primate cognition research. Rigorously controlled conditions that excluded social influences were the standard. The Wisconsin General Test Apparatus (WGTA), a setup in which a subject was isolated in a test cage and presented with an array of choices, was a widely adopted approach to studying the minds of other primates (Harlow, 1949; Suomi & Leroy, 1982). Although individual testing is still commonplace and does confer certain experimental advantages, researching primate cognition while subjects are in a social group can lend a number of advantages over individual testing.

Testing primates in a social environment that reflects their natural history maintains a more natural test environment that may be essential for an individual primate’s learning and typical cognitive performance (Drea, 2006; see also Koski & Burkart, 2015). For the large majority of primate species, this would be a social setting. Whereas individual testing may shed light on the cognitive capacities of primates, testing in a reduced social environment does little to inform us of the behaviors that would typically be expressed under natural social conditions and are thus subject to natural selection. Collaboration, prosociality, and social learning in particular appear to be affected by the presence of conspecifics, as well as the identity of the conspecifics and their relationship with the test subject, in many species of primates (reviewed in: Cronin, 2012; Yamamoto & Takimoto, 2012). In early studies, researchers typically dictated the social interactions of test subjects by deciding which animals to test in concert (e.g., Menzel Jr, Davenport & Rogers, 1972; Brosnan & de Waal, 2003; Horner et al., 2006). However, researchers are increasingly recognizing that testing primate cognition in the field or in less constrained social settings in captivity allows for subject-driven partner choice that provides a more valid representation of cognitive processes in several species (e.g., Whiten, Horner & de Waal, 2005; Noe, 2006; Crockford et al., 2012; Molesti & Majolo, 2016; Suchak et al., 2016). Maintaining the social environment that is characteristic of a species’ natural history as much as possible during cognitive testing improves the socio-ecological validity of the research. The increased validity should be especially pronounced for gregarious species and when social cognition is under investigation.

Even when non-social cognition is the focus of the research, removing primates from their familiar social setting may impact the validity of results via an increased stress response. Decreased stress is desirable not only to promote good animal welfare but also to assure validity of cognitive and behavioral research (Olsson & Westlund, 2007). There are likely cases in which subjects may experience less stress when isolated from conspecifics for cognitive testing. However, in general, for social species, separation from groupmates may induce negative physiological changes (e.g., Shively, Clarkson & Kaplan, 1989) whereas the presence of conspecifics may buffer individuals from psychological distress (e.g., Stanton, Patterson & Levine, 1985; Ragen et al., 2013). Separation-induced physiological changes may include the activation of the hypothalamic-pituitary-adrenal (HPA) axis (e.g., Gonzales, Coe & Levine, 1982; Dettmer et al., 2012), which has well-established influences on learning and cognition (McEwen & Sapolsky, 1995; Newcomer et al., 1999; Lyons et al., 2000; Song et al., 2006). Some captive facilities have gone to great measures to acclimate primates to routine separation from groupmates for testing (e.g., Hare, Call & Tomasello, 2001; Hopper et al., 2014), yet success may vary by species, individual temperaments, and the experimental protocol (Hopper et al., 2013). Because of these potential impacts of individual testing, which may confound results of such testing, we believe it is important to look to previously-run studies that have successfully tested primate cognition in a social setting to inform future experimental design of primate cognitive research. While we acknowledge that both social and individual testing have advantages and disadvantages, we believe highlighting social approaches is a useful exercise given that the default protocol for many research studies is to test primates individually or in pairs.

Although testing primates in a group setting has the potential to increase validity and minimize stress, such methods are often not readily adopted because of the logistical challenges and statistical limitations of social testing (e.g., Gazes et al., 2013). For example, when primates are tested in a social group in the wild or captivity, it can be difficult to elicit participation from all group members due to competition within the social group, interference from other group members, the ability to scrounge without participating, and/or the tendency of low-ranking individuals to abstain from participating in the presence of higher ranking others (e.g., Santos et al., 2002; Hopper et al., 2007). Another challenge is the reduced experimental control that often comes with social testing. It is not always practical to control design aspects such as the number of trials per individual, order of participation, nor the social composition of subgroups present (e.g., Crockford et al., 2012; House et al., 2014). Furthermore, it can be difficult and laborious to identify individual responses, either in real time or from video (e.g., Drea, 2006; van Leeuwen et al., 2013). Finally, statistically speaking, testing entire social groups may essentially reduce a researcher’s sample size, as the subjects within a single group often cannot be considered independent from one another (e.g., Burkart et al., 2014; Cronin et al., 2014a). This limitation is exacerbated by the fact that groups will rarely be uniform in size, demographic makeup, nor in their physical environment.

Here, we provide an overview of cognitive studies that have taken place with primates in a social setting over a sixteen-year period, since the year 2000. We do so in order to understand the frequency with which such testing occurs, how it is distributed across taxa and environments, and the range of sample sizes (social groups and individuals) that researchers have studied. Another aim of this survey is to extract potential solutions to the practical and statistical challenges of social testing. Given that increased validity and improved welfare may be gained from testing social species in a social setting (e.g., Drea, 2006; Koski & Burkart, 2015), we hope that by providing this information scientists may be better equipped with promising approaches for future research design.

## Survey Methodology

First, we used Google Scholar (https://scholar.google.com/) to systematically identify peer-reviewed journal articles (see Table S1). Searches were performed in English separately for each year between 2000 and 2015, inclusive, using the search term “primate AND cognition AND experiment AND social group.” Searches were performed in one year increments by the authors, completed in February of 2016. The resulting list of articles was then screened for whether the methods described non-human primates that were tested in groups comprised of three or more individuals. Given the difficulty of defining “cognition,” for our purposes we considered any research that included an experimental intervention and measured behavioral responses. Therefore, we also filtered by whether the publication included any kind of experimental manipulation (e.g., presented a stimulus or a task); studies that were purely observational were not considered here. We included a continuum of social testing settings, from open access to an apparatus for an entire social group (e.g., van de Waal, Claidière & Whiten, 2015) to cases in which individuals or dyads from a larger social group were tested separately but were not physically separated from a larger social group (for example if they could enter and exit a test booth through a swinging door as they chose, e.g., Fagot & Paleressompoulle, 2009). We excluded cases in which primates were tested socially but with non-conspecifics, except for one case in which naturally occurring mixed-species groups were studied in the field (Kirchhof & Hammerschmidt, 2006). Next, we then returned to each journal that contributed a result to the first round of searching and performed two supplemental searches. The first used the terms “primate AND playback” (because we noted several playback studies were missed in the broad Google Scholar search) and a second search using the terms “primate AND experiment AND cognition” (because we noted that several field studies did not use the term “social group” when studying primates in a social setting). We also included input from two external colleagues (EJC van Leeuwen & E van de Waal) and three reviewers of an earlier draft of this manuscript.

For each publication satisfying the above criteria, we extracted the following information and entered it into a database: institution name(s), test environment (field, zoo, laboratory, or sanctuary), species, number of social groups studied, number of individuals tested, keywords, and the full citation. Additionally, we noted for each publication whether the target information was reported in the manuscript (or supplemental information) exactly, as a range, or whether the information was unreported. For example, while some articles reported the exact number of individuals in the groups tested, others only reported the range of group sizes. To determine the median number of social groups and individuals studied per publication, we first filtered our results to include only those publications that reported this information (or a range across multiple groups, in which case the average was taken). Initially, we attempted to document the statistical approaches employed in each study but found we were unable to do so in a consistent way given the number of approaches used per study and variability in statistical reporting. The final database was not adjusted in any way to standardize the number of publications from a single author or site, but a bias toward overrepresentation from certain animals or institutions is considered in the Discussion. The database does not include information about studies that were attempted in a group setting but failed to be completed.

Disagreements or uncertainties about inclusion were decided on by all authors based on the contents of the publication and our criteria (publication authors were never contacted for clarification or additional information). Data were analyzed in R version 3.1.2 (R Core Team, 2014) and visualized using “ggplot2” (Wickham, 2009).

## Results

The search yielded 248 peer-reviewed publications in 43 journals (Table S1). The number of publications that measured primate cognition in a social setting increased significantly over the years examined (Spearman Rank Order correlation *r* = 0.80, *S* = 134.4, *P* < 0.001).

### Test environment

**Figure 1:**
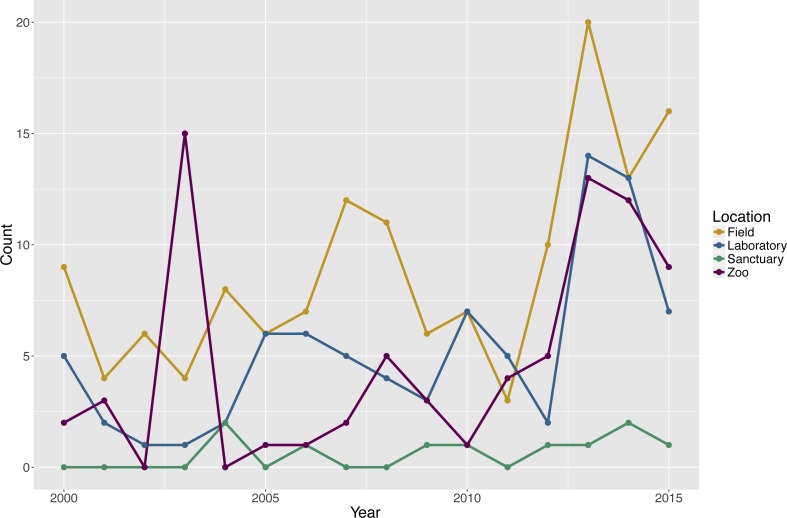
Count of publications testing primate cognition in a social setting, by environment and year of publication, with one entry per unique combination of environment type and species per publication.

The search revealed that 142 publications (57.3%) tested subjects in a field setting, 84 publications (33.9%) tested subjects in a laboratory setting, 76 publications (30.6%) tested subjects in a zoo setting, and 10 publications (4.0%) tested subjects in a sanctuary setting. Two publications did not report the setting for the testing of six groups. Note that the percentages sum to greater than 100% because some publications tested social groups in more than one type of environment and each unique species and environment combination within a publication was considered separately in the analyses (Fig. 1).

### Range of species tested

**Figure 2:**
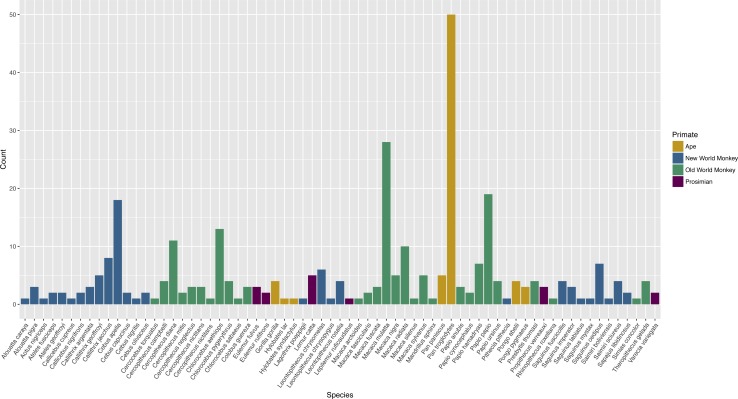
Count of publications testing primate cognition in a social setting, by species, with one entry per unique combination of environment type and species per publication.

The publications encompassed 71 different species (Table S1). Apes were tested in 25.4% of publications, Old World monkeys in 55.6% of publications, New World monkeys in 29.8%, and prosimians in 6.0% of publications. Twenty-six publications (10.5%) included subjects of more than one species. Chimpanzees (*Pan troglodytes*) were the most commonly tested species, represented in 18.5% of publications (Fig. 2).

### Number of subjects and social groups tested

Two hundred and twenty-one of the 248 publications (89.1%) provided information regarding the number of subjects tested. The median number of subjects tested per publication was 17 (range 1–335). Two hundred and eighteen of the publications (87.9%) provided information regarding the number of social groups tested. The median number of social groups tested per publication was 2 (range 1–55).

### Identification of promising approaches

We reviewed the literature for methodological approaches to overcoming four challenges common to social testing: reduced participation, reduced experimental control, difficulty identifying individual responses, and reduced sample size. All publications listed in Table S1 have overcome social testing challenges in some way, and may provide researchers with ideas for overcoming challenges specific to their own test environment or study species. However, to meet our aim of extracting potential solutions from the literature review that overcome these common practical and statistical challenges, we selected publications that we envisage will be useful to a broad audience. These publications are discussed in detail below.

## Discussion

Conducting cognitive experiments in a social group comes with several potential benefits, including testing animals in a context that reflects their natural history and improved animal welfare. However, the challenges posed, including reduced participation due to competition or aggression in the group, reduced experimental control, reduced effective sample size, and difficulty identifying individual responses, may lead researchers to be wary of engaging in social testing. In this review encompassing articles published since the year 2000, we found that the number of publications that have tested primate cognition in a social setting has steadily increased and the coverage of species, while not evenly distributed, has been broad. Furthermore, such experimental studies have taken place in a range of captive environments and in the field. These findings suggest that there are feasible approaches to studying cognition in a social setting that we can turn to to extract strategies to overcoming real and perceived research challenges with social testing.

### Strategies for overcoming the challenge of reduced participation

**Figure 3:**
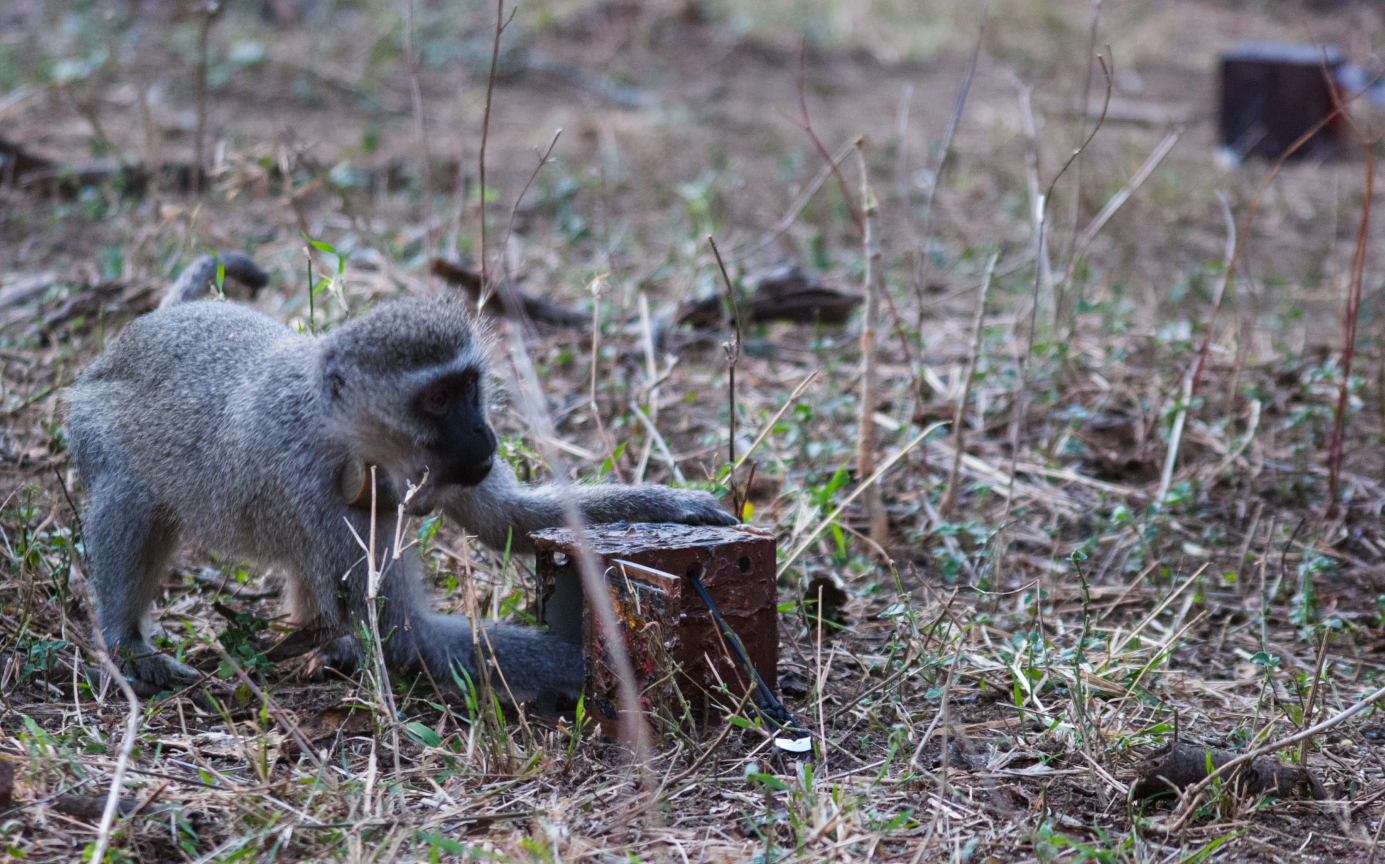
Vervet monkey accessing one of several available apparatuses at Inkawu Vervet Project, South Africa. Photo credit: Erica van de Waal.

A key challenge to testing primate cognition in a social setting is reduced participation due to competition or interference by group members, especially for lower-ranking individuals. Testing primates individually ensures that all subjects have equal exposure to the test stimuli and that their responses are uninfluenced by the actions or choices of others (Hopper et al., 2014). Hoewever, individual testing still does not guarantee that all subjects will participate, nor that their data are valid (Hopper et al., 2013). Therefore, we highlight four strategies that have been employed by researchers testing primates in a social setting to combat this. The first is to provide several test apparatuses so that access to them cannot be easily monopolized. For example, in a test of social learning in wild vervet monkeys (*Chlorocebus aethiops*), van de Waal and colleagues (2015) distributed between four and eight identical, baited puzzle boxes within the monkeys’ home range that allowed 40% of the individuals in the three wild groups under investigation to interact with the puzzle boxes (Fig. 3). The second strategy, which has been used with free-ranging rhesus macaques (*Macaca mulatta*) by Santos and colleagues, is to create a mobile task that experimenters can present to individuals or subgroups of interest as the opportunity arises (e.g., Santos, Hauser & Spelke, 2001; Hughes & Santos, 2012 Fig. 4; see also Almeling et al., 2016). The third strategy, designed to increase animals’ access to a single apparatus at a fixed location, is to run extended test sessions to allow lower-ranking individuals access to the test apparatus after more dominant group members become satiated. For example, Hopper et al. (2007) presented a tool-use task to captive chimpanzees (*Pan troglodytes*) in 5-hour long sessions, obtaining data from 79% of the chimpanzees in two captive groups. Our fourth suggestion comes from a new cognitive testing paradigm used with a group of zoo-housed Japanese macaques (*M. fuscata*), where the whole social group has access to touchscreens in two test booths at the periphery of their habitat when a researcher is present (Cronin, Hopper & Ross, 2015). The macaques were trained to recognize an ‘end-of-session’ cue that indicates they will not receive any additional rewards for the day, causing them to leave the testing booth and allowing untested individuals to participate. This has proven an effective way to obtain touchscreen trials from lower ranking individuals who participate once the higher-ranking ones have disengaged after receiving their end-of-session cue.

**Figure 4:**
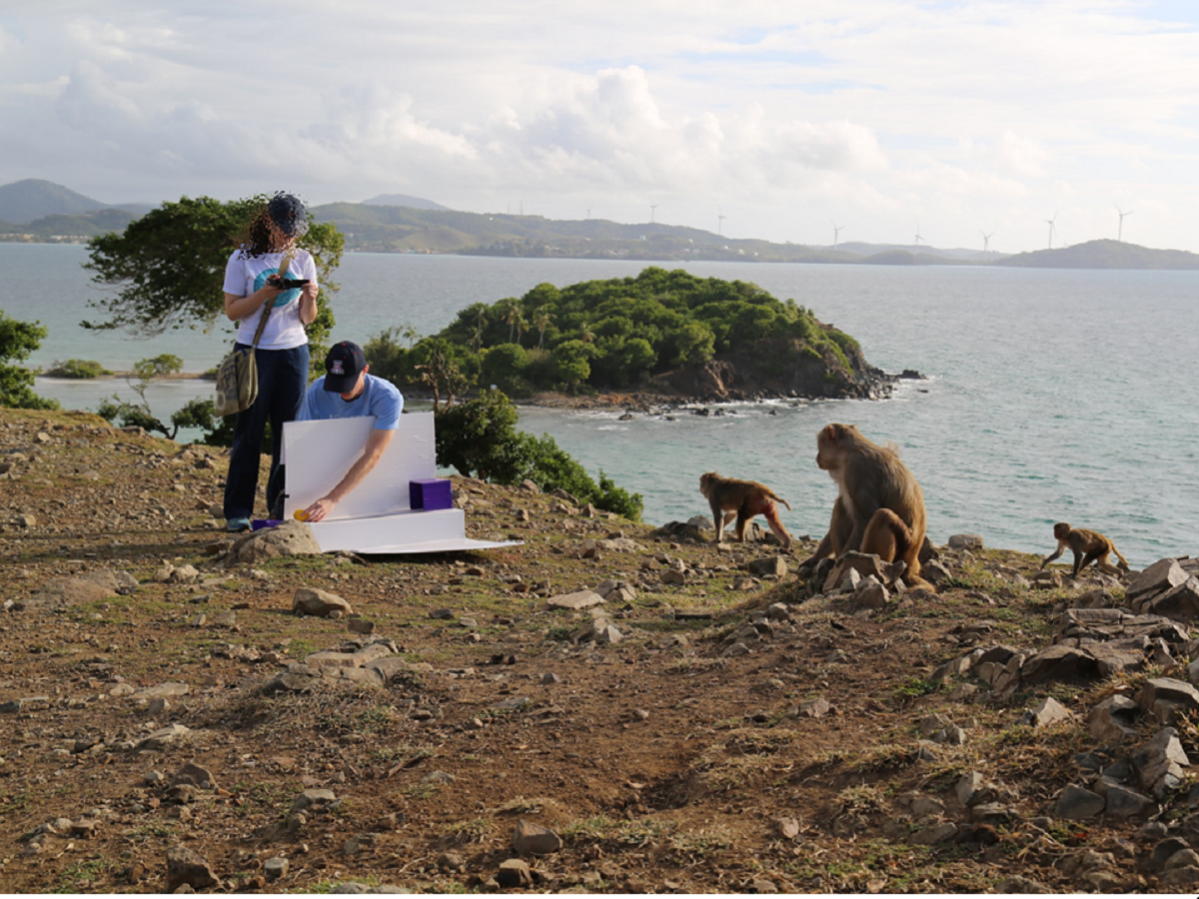
A portable experimental setup for testing rhesus macaque cognition at the Cayo Santiago Field Station, Puerto Rico. Photo credit: Alyssa Arre.

### Strategies for overcoming the challenge of reduced experimental control

A second challenge to testing primates in a social setting is reduced experimental control. Specifically, in a social setting it is not often practical to control experimental design aspects such as the number of trials per individual, order of participation, nor the social composition of subgroups present (Carter et al., 2014), as can be acheieved with experimenter-controlled pairings (e.g., Silk et al., 2005; Cronin et al., 2014b). One strategy for overcoming an unbalanced number of trials per subject is to set up the experiment and analysis such that proportions of response types (rather than frequencies) can be informative of the subject’s knowledge or preference. For example, House and colleagues (2014) tested prosocial behavior in several groups of captive chimpanzees without isolating subjects. They designed an apparatus that subjects could control at the perimeter of their enclosure and compared changes in the proportion of food-sharing attempts made by individuals when different food distributions were possible. By employing a within-subjects design, it was not necessary to obtain the same number of trials across all individuals or conditions (see also Finestone et al., 2014; Molesti & Majolo, 2016). Other dynamic aspects of the testing environment, such as the order of exposure, amount of previous experience with the task, or the current social composition, can now be handled statistically through the use of generalized mixed effects models when sample sizes are sufficient (see Crockford et al., 2012; House et al., 2014). Those studying social relationships or social learning may actively wish to test within-group dynamics using social network analyses (Whitehead, 2008), network-based diffusion analysis (Franz & Nunn, 2009; Franz & Nunn, 2010) or option-bias analysis (Kendal et al., 2010; Kendal et al., 2015). Mixed effects models are useful for handling datasets in which the same individuals are tested in different social configurations, and they can also handle non-factorial designs that may be prevalent in experiments that rely on opportunistic data collection in a captive or wild setting.

### Strategies for overcoming the challenge of identifying individual responses

**Figure 5:**
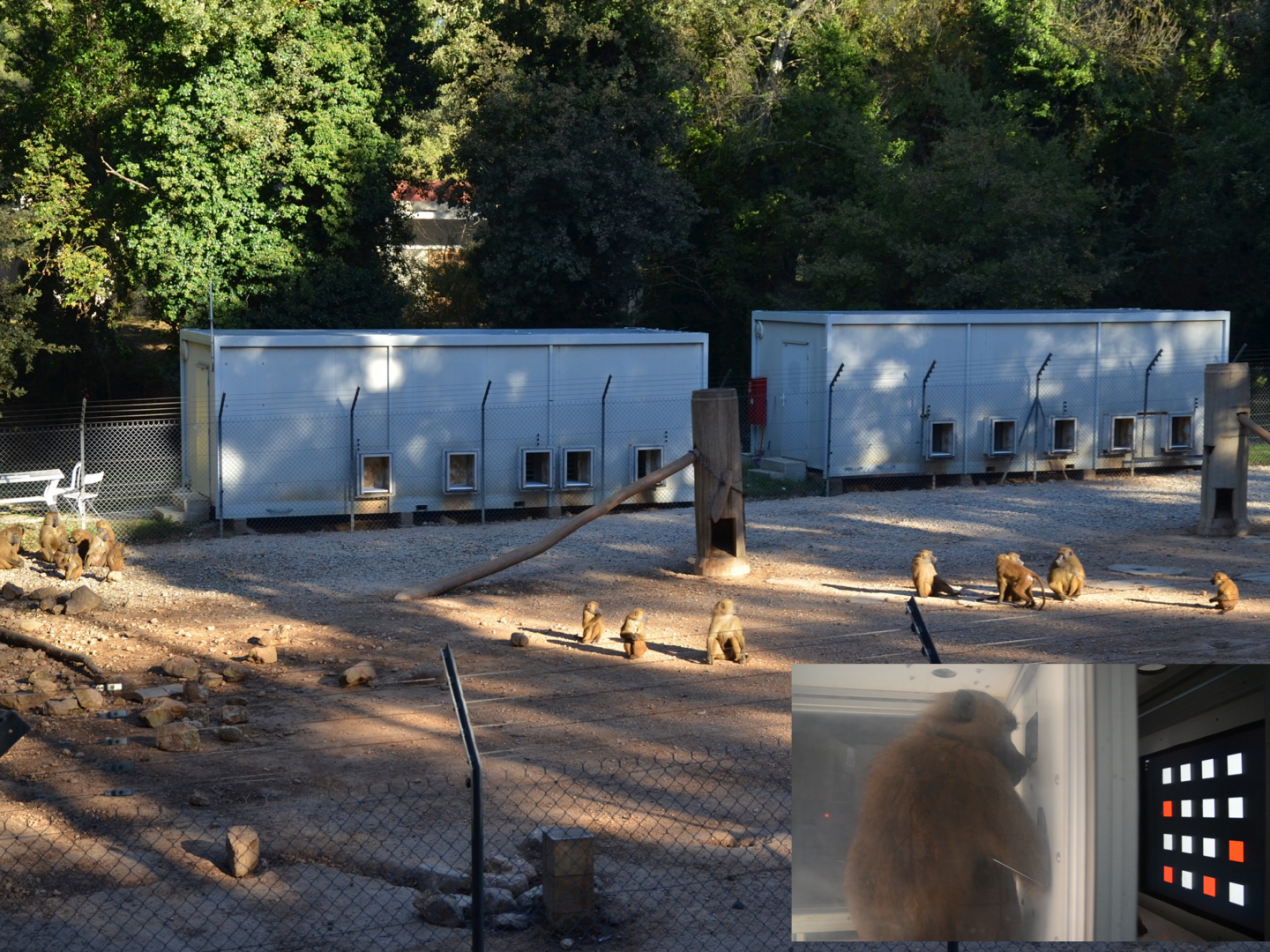
Guinea baboons with RFID chips to automate individual identification of responses in touchscreen booths at the CNRS Primate Center in Rousset-sur-Arc, France. Inset shows a baboon inside one of the touchscreen booths. Photo credit: Nicolas Claidière.

A third challenge to testing primate cognition in a social setting is the difficulty of identifying individual responses. If an animal is housed alone or removed from its social group for testing, researchers are relieved of the responsibility to track which individual is making a response (e.g., Basile & Hampton, 2017; Cantlon et al., 2015). Typically, when testing primates in a social setting, researchers video record experimental test sessions so that they can later code the individuals involved in a given trial (e.g., van Leeuwen et al., 2013). Although a commonly-used and successful method, video recording and later coding experiments is a time-consuming process that is made more complex with small-bodied and fast-moving primates, or when testing animals in the field where viewing animals may be more difficult (e.g., Gunhold, Whiten & Bugnyar, 2014). More recently, creative testing arenas in captive settings have enabled researchers to identify individual subjects without removing the animal from its home enclosure. Researchers in zoos and laboratories have designed touchscreen test stations within, or attached to the periphery of, primates’ home enclosures, that allow animals to participate individually, while not being socially isolated from their group (Fagot & Paleressompoulle, 2009; Whitehouse et al., 2013; Cronin, Hopper & Ross, 2015). Live-stream footage or video recording can track which individuals are in the booth at any time, or, if resources allow, radio frequency identification (RFID) chips can be used to automatically track the identity of participating individuals and even queue up individually tailored task sets (Fagot & Paleressompoulle, 2009; Gazes et al., 2013; Claidière et al., 2014; Fig. 5). The Primate Research Institute at Kyoto University utilizes an automated face recognition program that allows them to identify the chimpanzee when he or she voluntarily breaks off from the social group to engage in touchscreen research. A system such as this also allows for automated coding of the participant’s identity and personalized administration of tasks.

### Strategies for overcoming the challenge of reduced sample size

A fourth challenge to testing in a social setting to address is the reduction in sample size. Given that individuals within a group are not independent from one another, the conservative statistical approach often used is to consider sample size as equivalent to social group. Obviously this is a limitation of statistical power and may turn researchers away from testing primates socially. However, as mentioned above, statistical mixed effects models can help with issues of interdependence through the inclusion of individuals and groups as random factors (Schneider, Melis & Tomasello, 2012; Gunhold, Whiten & Bugnyar, 2014). Furthermore, many research groups have included several social groups in a single publication, and we encourage others to follow their lead when feasible (e.g., Whiten et al., 2007; Burkart et al., 2014). It is common for those running experimental studies with wild primates to only work with individuals from one or two groups (median number of social groups in this analysis was two), yet several field sites have now been established that include multiple social groups (e.g., Cayo Santiago, Puerto Rico, Santos, Hauser & Spelke, 2001; Inkawu Vervet Project, South Africa, van de Waal, Borgeaud & Whiten, 2013). One way forward may be to seek out research at locations that are home to several social groups of the same species (e.g., as is typical of primate sanctuaries or National Primate Research Centers). Lastly, although numbers may be small at a single institution, research in zoos where collaboration between institutions is often already in the culture may be a feasible route to obtaining data from several groups and species (e.g., Dean et al., 2011; Cronin, 2017). Researchers would need to consider variation introduced from multiple sites, but would likely increase their statistical power and the generalizability of their findings.

## Conclusions

In addition to illuminating strategies for overcoming challenges of group testing, this literature review revealed a number of interesting trends from sixteen years of research. First, the knowledge of primate cognition gained from testing primates in a social setting is not evenly distributed across species, with nearly a fifth of publications focusing on chimpanzees. A disproportionate emphasis on a small number of species has been noted previously as a criticism of comparative psychological research (e.g., Beach, 1950; Beran et al., 2014) and remains a pervasive issue for primate cognition research. It is likely that an over-emphasis on chimpanzees has influenced the inferences drawn about the unique nature of human cognition and the evolution of cognition more generally (e.g., Melis & Warneken, 2016). At a broader taxonomic level, prosimians were the least represented (approximately one in twenty publications included prosimians). This may reflect an under-representation of prosimians in cognitive research (Melfi, 2005), however studies of species naturally found living solitary or in pairs, as is the case for orangutans, night monkeys, and several prosimian species, would not have been included here, as discussed below.

There were several practical decisions that limited the scope of this review. The first is that we restricted the search to research with non-human primates. However, we hope some lessons learned here may apply to other social species as well. In fact, recent work has called to light the limited validity of testing human cognition in non-social paradigms as well (e.g., Reader & Holmes, 2016). The selection criteria included studies that took place in social groups of three or more, primarily to avoid studies using the common approach of testing dyads extracted from a larger group (e.g., Melis, Hare & Tomasello, 2006; Cronin et al., 2014b; Brosnan et al., 2015), as this approach misses the spirit of what we were trying to evaluate. Admittedly, this leaves species naturally living alone or in pairs to be excluded from our dataset, which may affect our conclusions above about the relative contribution of species to studies of cognition in a social setting. We also opted to include only studies in which there was some experimental manipulation or presentation of a task in order to have objective criteria for what was included as experimental, “cognitive research.” This ignores several interesting findings from observational research relevant to primate cognition, such as vocal learning and cultural transmission of naturally-occurring behaviors (e.g., Luncz, Mundry & Boesch, 2012; van Leeuwen, Cronin & Haun, 2014). With the focus on experimental, cognitive research, we also did not tackle other realms of primate research, such as biomedical research, an area that may also be growing in its desire to test socially-housed primates for the same reasons of increased validity and improved welfare (e.g., Kelly, 2008; DiVincenti & Wyatt, 2011). Unfortunately, due to practical limitations, this review also excludes research that was not published in English.

It is also worth noting that this review did not provide a direct comparison of individual versus group testing; our aim was to highlight practical take-aways from previously-published work to facilitate future cognitive testing of primates in a social setting. Indeed, such a comparative evaluation would be difficult. There are very few examples in which primates have been tested with the same apparatus in a social as well as an individual setting to test the effect of social environment on cognition (although see Hopper et al., 2015b, and also Krasheninnikova & Schneider, 2014 for an example with birds, *Amazona amazonica*). Typically, if an experiment tests both individuals and groups of primates with the same task, the individually-tested subjects represent ‘control’ animals to be compared to socially-tested subjects, in studies of social learning for example (e.g., Whiten, Horner & de Waal, 2005; Kendal et al., 2015). However, the relative impact of the social environment on differences in learning are rarely considered.

The majority of publications describing tests of primates in social groups resulted from experiments that took place in the field and laboratories; these environments were nearly equally represented. Zoos and sanctuaries, on the other hand, contributed only a small fraction of the publications. Notably, representation of environments in our database is characterized by number of publications, not by number of institutions. Moving forward, zoos and sanctuaries may provide a valuable resource for conducting enriching, non-invasive, voluntary research with socially-housed primates (Cronin, 2017; Hopper, 2017), and this may be especially attractive to institutions if the research proves enriching to the subjects (Hopper, Shender & Ross, 2016). In the past sixteen years, there have been over two hundred publications describing cognitive research with primate subjects tested in the presence of at least two other conspecifics. These studies spanned several species and test environments. We also note that, although the majority of studies identified in our review, unsurprisingly, tested aspects of primate social cognition (e.g., social learning, cooperation, competition, and communication), this was not exclusively so. Highlighting that a social test setting does not prohibit the testing of non-social cognition, our review revealed tests of, for example, analogical reasoning (Fagot & Maugard, 2013), memory (Fagot & de Lillo, 2011; Maugard, Marzouki & Fagot, 2013), visual perception (Cheries et al., 2006), numerical understanding (Hauser, Carey & Hauser, 2000), and personality (Carter et al., 2012; Neumann et al., 2013), although we note the inherent complexity of teasing apart “social” from “non-social” cognition (c.f. Seyfarth & Cheney, 2017).

Many researchers are overcoming several of the hurdles inherent to social testing using creative paradigms, embracing collaboration, and employing advances in statistics and technology. Moving forward, we hope that researchers will consider the strategies we have highlighted as well as publications summarized in Table S1 as a resource for designing, conducting and analyzing cognitive research with primates in a social setting. Social testing will continue to present challenges; many of the strategies highlighted above require additional time, collaboration, and statistical expertise. However, continuing to focus efforts on facilatiting cognitive testing in a social setting should continue to strengthen the validity of conclusions drawn from studies of primate cognition and improve animal welfare.

##  Supplemental Information

10.7717/peerj.3649/supp-1 Supplemental Information 1 PRISMA (2009) Checklist for Systematic Reviews

10.7717/peerj.3649/supp-2 Table S1 Published experiments of primate cognition in a social setting (2000-2015) The publications that reported tests of primate cognition in a social setting published between 2000 and 2015 (inclusive), sorted by species, with one entry per unique combination of environment type and species per publication.

## Additional Information and Declarations

### Data Availability

The following information was supplied regarding data availability: The raw data has been supplied as a Supplemental File.
