# Supplementary material for: Studying primate cognition in a social setting to improve validity and welfare: a literature review highlighting successful approaches
Source: PeerJ. 2017 Aug 3;5:e3649. doi: 10.7717/peerj.3649 (PMC5545107; doi:10.7717/peerj.3649)
Supplement: Table S1 — The publications that reported tests of primate cognition in a social setting published between 2000 and 2015 (inclusive), sorted by species, with one entry per unique combination of environment type and species per publication. [file peerj-05-3649-s002.pdf]

Table S1. The publications that tested primate cognition in a social setting published between 2000 and 2015 (inclusive), with one entry per unique combination of environment type and species per publication.

Species	Reference	Title of Article	Environment Type	Number of Groups Tested	Number of Individuals Tested
Alouatta caraya	da Cunha et al., 2006	Roars of black howler monkeys (*Alouatta caraya*): evidence for a function in inter-group spacing	field	1	13
Alouatta pigra	Briseno-Jamarillo et al., 2015	Individual voice recognition and an auditory map of neighbours in free-ranging black howler monkeys (*Alouatta pigra*)	field	6	45
Alouatta pigra	Kitchen et al., 2004	Subordinate male black howler monkey (*Alouatta pigra*) responses to loud calls: experimental evidence for the effects of intra-group male relationships and age	sanctuary	NA	10
Alouatta pigra	Kitchen, 2004	Alpha male black howler monkey responses to loud calls: effect of numeric odds, male companion behavior and reproductive investment	sanctuary	NA	12
Alouatta pigra	Kitchen, 2006	Experimental test of female black howler monkey (*Alouatta pigra*) responses to loud calls from potentially infanticidal males: effects of numeric odds, vulnerable offspring, and companion behavior	sanctuary	12	27
Aotus nigriceps	Bicca-Marques & Garber, 2004	Use of spatial, visual, and olfactory information during foraging in wild nocturnal and diurnal anthropoids: A field experiment comparing Aotus, Callicebus, and Saguinus	field	1	4
Ateles fusciceps	Nelson & Boeving, 2015	Precise digit use increases the expression of handedness in Colombian spider monkeys (*Ateles fusciceps rufiventris*)	zoo	1	9
Ateles fusciceps	Nelson et al., 2015	Evaluating handedness measures in spider monkeys	zoo	1	5
Ateles geoffroyi	Burkart et al., 2014	The evolutionary origin of human hyper-cooperation	zoo	2	23
Callicebus cupreus	Bicca-Marques & Garber, 2004	Use of spatial, visual, and olfactory information during foraging in wild nocturnal and diurnal anthropoids: A field experiment comparing *Aotus, Callicebus*, and *Saguinus*	field	1	5
Callicebus nigrifrons	Cäsar et al., 2013	Titi monkey call sequences vary with predator location and type	field	5	NA
Callicebus nigrifrons	Caselli et al., 2015	Playback responses of socially monogamous black-fronted titi monkeys to simulated solitary and paired intruders	field	5	12
Callithrix argentata	Day et al., 2003	Neophilia, innovation and social learning: A study of intergeneric differences in callitrichid monkeys	zoo	3	22
Callithrix geoffroyi	Braccini & Caine, 2009	Hand preference predicts reactions to novel foods and predators in marmosets (*Callithrix geoffroyi*)	zoo	2	18
Callithrix geoffroyi	Day et al., 2003	Neophilia, innovation and social learning: A study of intergeneric differences in callitrichid monkeys	zoo	3	15
Callithrix geoffroyi	Kitzmann & Caine, 2009	Marmoset (*Callithrix geoffroyi*) food-associated calls are functionally referential	zoo	2	12
Callithrix geoffroyi	Petracca & Caine, 2013	Alarm calls of marmosets (*Callithrix geoffroyi*) to snakes and perched raptors	zoo	2	12
Callithrix jacchus	Burkart & van Schaik, 2013	Group service in macaques (*Macaca fuscata*), capuchins (*Cebus apella*) and marmosets (*Callithrix jacchus*): A comparative approach to identifying proactive prosocial motivations	laboratory	1	7
Callithrix jacchus	Burkart et al., 2014	The evolutionary origin of human hyper-cooperation	laboratory	5	30
Callithrix jacchus	Gunhold et al., 2014	Memory, transmission and persistence of alternative foraging techniques in wild common marmosets	field	13	111

Table S1. The publications that tested primate cognition in a social setting published between 2000 and 2015 (inclusive), with one entry per unique combination of environment type and species per publication.

Species	Reference	Title of Article	Environment Type	Number of Groups Tested	Number of Individuals Tested
Callithrix jacchus	Gunhold, Whiten & Bugnyar, 2014	Video demonstrations seed alternative problem-solving techniques in wild common marmosets	field	12	109
Callithrix jacchus	Halsey et al., 2006	Can wild common marmosets (*Callithrix jacchus*) solve the parallel strings task?	field	2	13
Callithrix jacchus	Pesendorfer et al., 2009	The maintenance of traditions in marmosets: individual habit, not social conformity? A field experiment	field	9	36
Callithrix jacchus	Schiel & Huber, 2006	Social influences on the development of foraging behavior in free-living common marmosets (*Callithrix jacchus*)	field	4	32
Callithrix jacchus	Watson, Buchanan-Smith & Caldwell, 2014	Call playback artificially generates a temporary cultural style of high affiliation in marmosets	laboratory	19	31
Cebus apella	Burkart & van Schaik, 2013	Group service in macaques (*Macaca fuscata*), capuchins (*Cebus apella*) and marmosets (*Callithrix jacchus*): A comparative approach to identifying proactive prosocial motivations	zoo	1	7
Cebus apella	Burkart et al., 2014	The evolutionary origin of human hyper-cooperation	laboratory	2	17
Cebus apella	Burkart et al., 2014	The evolutionary origin of human hyper-cooperation	zoo	1	8
Cebus apella	Burkart et al., 2014	The evolutionary origin of human hyper-cooperation	laboratory	2	17
Cebus apella	Burkart et al., 2014	The evolutionary origin of human hyper-cooperation	zoo	1	8
Cebus apella	Crast, Hardy & Fragaszy, 2010	Inducing traditions in captive capuchin monkeys (*Cebus apella*)	laboratory	6	27
Cebus apella	Dean et al., 2012	Identification of the social and cognitive processes underlying human cumulative culture	laboratory	1	22
Cebus apella	Di Bitetti, 2003	Food-associated calls of tufted capuchin monkeys (*Cebus apella nigritus*) are functionally referential signals	field	1	NA
Cebus apella	Dindo, Whiten & de Waal, 2009	In-group conformity sustains different foraging traditions in capuchin monkeys (*Cebus apella*)	laboratory	2	27
Cebus apella	Evans & Westergaard, 2006	Self-control and tool use in tufted capuchin monkeys (*Cebus apella*)	laboratory	4	20
Cebus apella	Flemming, Rattermann & Thompson, 2006	Differential individual access to and use of reaching tools in social groups of capuchin monkeys (*Cebus apella*) and human infants (*Homo sapiens*)	laboratory	1	6
Cebus apella	Janson, 2007	Experimental evidence for route integration and strategic planning in wild capuchin monkeys	field	1	32.5
Cebus apella	Morton et al., 2013	Taking personality selection bias seriously in animal cognition research: a case study in capuchin monkeys (*Sapajus apella*)	zoo	2	18
Cebus apella	Wheeler & Hammerschmidt, 2013	Proximate factors underpinning receiver responses to deceptive false alarm calls in wild tufted capuchin monkeys: is it counterdeception?	field	NA	NA
Cebus apella	Wheeler et al., 2014	Competition-induced stress does not explain deceptive alarm calling in tufted capuchin monkeys	field	2	45
Cebus apella	Wheeler, 2008	Selfish or altruistic? An analysis of alarm call function in wild capuchin monkeys, *Cebus apella nigritus*	field	3	49.5
Cebus apella	Wheeler, 2009	Monkeys crying wolf? Tufted capuchin monkeys use anti-predator calls to usurp resources from conspecifics	field	1	26

Table S1. The publications that tested primate cognition in a social setting published between 2000 and 2015 (inclusive), with one entry per unique combination of environment type and species per publication.

**Species**	**Reference**	**Title of Article**	**Environment Type**	**Number of Groups Tested**	**Number of Individuals Tested**
Cebus apella	Wheeler, 2010	Decrease in alarm call response among tufted capuchins in competitive feeding contexts: possible evidence for counterdeception	field	1	8
Cebus apella	Wheeler, 2010	Production and perception of situationally variable alarm calls in wild tufted capuchin monkeys (*Cebus apella nigritus*)	field	NA	NA
Cebus capucinus	Meno et al., 2013	Development of snake-directed antipredator behavior by wild white-faced capuchin monkeys: II Influence of the social environment	field	6	15
Cebus capucinus	Meunier, Petit & Deneubourg, 2008	Social facilitation of fur rubbing behavior in white-faced capuchins	laboratory	1	12
Cebus nigritis	Gomes & Bicca-Marques, 2012	Capuchin monkeys use spatial and visual information during within patch foraging	field	1	8
Cebus olivaceus	Dubois et al., 2000	Location-specific responsiveness to environmental perturbations in wedge-capped capuchins (*Cebus olivaceus*)	laboratory	1	5
Cebus olivaceus	Dubois et al., 2001	Spatial facilitation in a probing task in wedge-capped capuchins (*Cebus olivaceus*)	laboratory	1	4
Cebus apella	Addessi & Visalberghi, 2001	Social facilitation of eating novel food in tufted capuchin monkeys (*Cebus apella*): input provided by group members and responses affected in the observer	laboratory	2	15
Cercocebus torquatus	Candiotti et al., 2013	Voice discrimination in four primates	laboratory	1	6
Cercopithecus campbelli	Candiotti et al., 2013	Voice discrimination in four primates	laboratory	1	8
Cercopithecus campbelli	Lemasson et al., 2005	Socially meaningful vocal plasticity in adult Campbell's monkeys (*Cercopithecus campbelli*)	laboratory	1	15
Cercopithecus campbelli	Ouattara et al., 2009	The alarm call system of female Campbell's monkeys	field	7	NA
Cercopithecus campbelli	Zuberbühler, 2001	Predator-specific alarm calls in Campbell's monkeys, *Cercopithecus campbelli*	field	55	NA
Cercopithecus diana	Bshary, 2001	Diana monkeys, *Cercopithecus diana*, adjust their anti-predator response behaviour to human hunting strategies	field	NA	NA
Cercopithecus diana	Coye et al., 2015	Suffixation influences receivers' behaviour in non-human primates	field	42	NA
Cercopithecus diana	Stephan & Zuberbühler, 2008	Predation increases acoustic complexity in primate alarm calls	field	NA	NA
Cercopithecus diana	Stephan & Zuberbühler, 2008	Predation increases acoustic complexity in primate alarm calls	field	NA	NA
Cercopithecus diana	Stephan & Zuberbühler, 2014	Predation affects alarm call usage in female Diana monkeys (*Cercopithecus diana diana*)	field	NA	NA
Cercopithecus diana	Stephan & Zuberbühler, 2014	Predation affects alarm call usage in female Diana monkeys (*Cercopithecus diana diana*)	field	NA	NA
Cercopithecus diana	Zuberbühler, 2000	Referential labelling in Diana monkeys	field	23	23
Cercopithecus diana	Zuberbühler, 2000	Interspecies semantic communication in two forest primates	field	NA	NA
Cercopithecus diana	Zuberbühler, 2000	Causal knowledge of predators' behaviour in wild Diana monkeys	field	50	NA

Table S1. The publications that tested primate cognition in a social setting published between 2000 and 2015 (inclusive), with one entry per unique combination of environment type and species per publication.

**Species**	**Reference**	**Title of Article**	**Environment Type**	**Number of Groups Tested**	**Number of Individuals Tested**
Cercopithecus diana	Zuberbühler, 2000	Causal cognition in a non-human primate: field playback experiments with Diana monkeys	field	50	NA
Cercopithecus diana	Zuberbühler, 2002	A syntactic rule in forest monkey communication	field	NA	NA
Cercopithecus mitis	Murphy et al., 2013	Male blue monkey alarm calls encode predator type and distance	field	NA	NA
Cercopithecus mitis	Papworth et al., 2008	Male blue monkeys alarm call in response to danger experienced by others	field	34	34
Cercopithecus neglectus	Candiotti et al., 2013	Voice discrimination in four primates	laboratory	3	4
Cercopithecus neglectus	Candiotti et al., 2013	Voice discrimination in four primates	zoo	2	7
Cercopithecus neglectus	Candiotti et al., 2013	Voice discrimination in four primates	zoo	1	2
Cercopithecus nictitans	Arnold & Zuberbühler, 2006	The alarm-calling system of adult male putty-nosed monkeys, *Cercopithecus nictitans martini*	field	NA	NA
Cercopithecus nictitans	Arnold & Zuberbühler, 2008	Meaningful call combinations in a non-human primate	field	1	17
Cercopithecus nictitans	Arnold & Zuberbühler, 2013	Female putty-nosed monkeys use experimentally altered contextual information to disambiguate the cause of male alarm calls	field	1	NA
Cercopithecus nictitans	Arnold et al., 2008	A forest monkey's alarm call series to predator models	field	NA	NA
Chlorocebus aethiops	Borgeaud et al., 2015	Age/sex differences in third-party rank relationship knowledge in wild vervet monkeys, *Chlorocebus aethiops pygeruthrus*	field	3	35
Chlorocebus aethiops	Borgeaud, van de Waal & Bshary, 2013	Third-party ranks knowledge in wild vervet monkeys (*Chlorocebus aethiops pygerythrus*)	field	3	16
Chlorocebus aethiops	James et al., 2007	Dimensions of impulsivity are associated with poor spatial working memory performance in monkeys	laboratory	2	18
Chlorocebus aethiops	van de Waal & Bshary, 2011	Social learning abilities of wild vervet monkeys in a two-step task artificial fruit experiment	field	6	28
Chlorocebus aethiops	van de Waal & Whiten, 2012	Spontaneous emergence, imitation and spread of alternative foraging techniques among groups of vervet monkeys	field	4	51
Chlorocebus aethiops	van de Waal et al., 2010	Selective attention to philopatric models causes directed social learning in wild vervet monkeys	field	6	64
Chlorocebus aethiops	van de Waal et al., 2012	Similarity in food cleaning techniques within matrilines in wild vervet monkeys	field	6	63
Chlorocebus aethiops	van de Waal et al., 2015	Wild vervet monkeys copy alternative methods for opening an artificial fruit	field	3	121
Chlorocebus aethiops	van de Waal, Borgeaud & Whiten, 2013	Potent social learning and conformity shape a wild primate's foraging decisions	field	4	42
Chlorocebus aethiops	van de Waal, Bshary & Whiten, 2014	Wild vervet monkey infants acquire the food-processing variants of their mothers	field	4	17
Chlorocebus aethiops	van de Waal, Claidière & Whiten, 2013	Social learning and spread of alternative means of opening an artificial fruit in four groups of vervet monkeys	field	4	34

Table S1. The publications that tested primate cognition in a social setting published between 2000 and 2015 (inclusive), with one entry per unique combination of environment type and species per publication.

**Species**	**Reference**	**Title of Article**	**Environment Type**	**Number of Groups Tested**	**Number of Individuals Tested**
Chlorocebus aethiops	van de Waal, Claidière & Whiten, 2014	Wild vervet monkeys copy alternative methods for opening an artificial fruit	field	3	64
Chlorocebus pygerythrus	Borgeaud & Bshary, 2015	Wild vervet monkeys trade tolerance and specific coalitionary support for grooming in experimentally induced conflicts	field	3	17
Chlorocebus pygerythrus	Burns et al., 2013	Barbados green monkeys (*Chlorocebus sabaeus*) recognize ancestral alarm calls after 350 years of isolation	field	1	15
Chlorocebus pygerythrus	Fruteau, van Damme & Noë, 2013	Vervet monkeys solve a multiplayer ''Forbidden Circle Game'' by queuing to learn restraint	field	2	14
Chlorocebus pygerythrus	Noe & Laporte, 2014	Socio-spatial cognition in vervet monkeys	field	2	26
Chlorocebus sabaeus	Price & Fischer, 2014	Meaning attribution in the West African green monkey: influence of call type and context	field	4	36
Chlorocebus sabeus	Fruteau, van Damme & Noë, 2013	Vervet monkeys solve a multiplayer ''Forbidden Circle Game'' by queuing to learn restraint	laboratory	1	25
Colobus guereza	Candiotti et al., 2013	Voice discrimination in four primates	zoo	1	7
Colobus guereza	Price & Caldwell, 2007	Artificially generated cultural variation between two groups of captive monkeys, *Colobus guereza kikuyuensis*	zoo	2	4
Colobus guereza	Schel et al., 2010	Predator-deterring alarm call sequences in Guereza colobus monkeys are meaningful to conspecifics	field	NA	NA
Eulemur fulvus	Fichtel & Hammerschmidt, 2002	Responses of redfronted leumurs to experimentally modified alarm calls: evidence for urgency-based changes in call structure	field	3	9
Eulemur fulvus	Fichtel & Kappeler, 2002	Anti-predator behavior of group-living Malagasy primates: mixed evidence for a referential alarm call system	field	4	6
Eulemur fulvus	Fichtel, 2004	Reciprocal recognition of sifaka (*Propithecus verrauxi verreauxi*) and redfronted lemur (*Eulemur fulvus rufus*) alarm calls	field	4	8
Eulemur rufifrons	Huebner & Fichtel, 2015	Innovation and behavioral flexibility in wild redfronted lemurs (*Eulemur rufifrons*)	field	4	29
Eulemur rufifrons	Schnoell & Fichtel, 2012	Wild redfronted lemurs (*Eulemur rufifrons*) use social information to learn new foraging techniques	field	4	37
Gorilla gorilla	Lonsdorf et al., 2009	An experimental, comparative investigation of tool use in chimpanzees and gorillas	zoo	2	8
Gorilla gorilla	Parron et al., 2008	Behavioural responses to photographs by pictorially naïve baboons (*Papio anubis*), gorillas (*Gorilla gorilla*) and chimpanzees (*Pan troglodytes)*	zoo	NA	2
Gorilla gorilla	Parron et al., 2008	Behavioural responses to photographs by pictorially naïve baboons (*Papio anubis*), gorillas (*Gorilla gorilla*) and chimpanzees (*Pan troglodytes)*	zoo	NA	2
Gorilla gorilla	Pitman & Shumaker, 2009	Does early care affect joint attention in great apes (*Pan troglodytes, Pan paniscus, Pongo abelii, Pongo pygmaeus, Gorilla gorilla*)?	unknown	NA	7
Hylobates lar	Burkart et al., 2014	The evolutionary origin of human hyper-cooperation	zoo	1	3
Hylobates lar	Burkart et al., 2014	The evolutionary origin of human hyper-cooperation	zoo	1	3

Table S1. The publications that tested primate cognition in a social setting published between 2000 and 2015 (inclusive), with one entry per unique combination of environment type and species per publication.

**Species**	**Reference**	**Title of Article**	**Environment Type**	**Number of Groups Tested**	**Number of Individuals Tested**
Hylobates syndactylus	Burkart et al., 2014	The evolutionary origin of human hyper-cooperation	zoo	1	3
Hylobates syndactylus	Burkart et al., 2014	The evolutionary origin of human hyper-cooperation	zoo	1	3
Lagothrix poeppigii	Papworth et al., 2013	Hunted wooly monkeys (*Lagothrix poeppigii*) show threat-sensitive responses to human presence	field	7	10
Lemur catta	Burkart et al., 2014	The evolutionary origin of human hyper-cooperation	laboratory	1	8
Lemur catta	Burkart et al., 2014	The evolutionary origin of human hyper-cooperation	laboratory	1	8
Lemur catta	Kendal et al., 2010	Evidence for social learning in wild lemurs (*Lemur catta*)	field	2	28
Lemur catta	Kulahci et al., 2015	Lemurs groom-at-a-distance through vocal networks	field	2	38
Lemur catta	Kulahci et al., 2015	Lemurs groom-at-a-distance through vocal networks	laboratory	2	15
Lemur catta	Nunn & Deaner, 2004	Patterns of participation and free riding in territorial conflicts among ringtailed lemurs (*Lemur catta*)	laboratory	2	14
Leontopithecus chrysomelas	Burkart et al., 2014	The evolutionary origin of human hyper-cooperation	laboratory	1	6
Leontopithecus chrysomelas	Burkart et al., 2014	The evolutionary origin of human hyper-cooperation	laboratory	1	6
Leontopithecus chrysomelas	Day et al., 2003	Neophilia, innovation and social learning: A study of intergeneric differences in callitrichid monkeys	zoo	3	12
Leontopithecus chrysomelas	de A Moura et al., 2010	Food sharing in lion tamarins (*Leontopithecus chrysomelas*): does foraging difficulty affect investment in young by breeders and helpers?	unknown	5	24
Leontopithecus chrysomelas	Rapaport, 2001	Food transfer among adult lion tamarins: Mutualism, reciprocity or one-sided relationships?	zoo	1	5
Leontopithecus chrysopygus	Day et al., 2003	Neophilia, innovation and social learning: A study of intergeneric differences in callitrichid monkeys	zoo	2	13
Leontopithecus rosalia	Day et al., 2003	Neophilia, innovation and social learning: A study of intergeneric differences in callitrichid monkeys	zoo	3	28
Leontopithecus rosalia	Rapaport, 2001	Food transfer among adult lion tamarins: Mutualism, reciprocity or one-sided relationships?	zoo	4	16
Lepilemur ruficaudatus	Fichtel, 2007	Avoiding predators at night: Antipredator strategies in red-tailed sportive lemurs (*Lepilemur ruficaudatus*)	field	7	7
Macaca arctoides	Paukner & Anderson, 2006	Video-induced yawning in stumptail macaques (*Macaca arctoides*)	laboratory	5	22
Macaca fascicularis	Gygax, 2000	Hiding behaviour of long-tailed macaques (*Macaca fascicularis*): II Use of hiding places during aggressive interactions	laboratory	1	30
Macaca fascicularis	Toxopeus et al., 2005	Effects of trait anxiety on performance of socially housed monkeys in a learning test	laboratory	2	15
Macaca fuscata	Burkart & van Schaik, 2013	Group service in macaques (*Macaca fuscata*), capuchins (*Cebus apella*) and marmosets (*Callithrix jacchus*): A comparative approach to identifying proactive prosocial motivations	zoo	1	10

Table S1. The publications that tested primate cognition in a social setting published between 2000 and 2015 (inclusive), with one entry per unique combination of environment type and species per publication.

**Species**	**Reference**	**Title of Article**	**Environment Type**	**Number of Groups Tested**	**Number of Individuals Tested**
Macaca fuscata	Burkart et al., 2014	The evolutionary origin of human hyper-cooperation	zoo	1	11
Macaca fuscata	Burkart et al., 2014	The evolutionary origin of human hyper-cooperation	zoo	1	11
Macaca fuscata	Shizawa et al., 2005	Playback experiment to test maternal responses of Japanese macaques (*Macaca fuscata*) to their own infant's call when the infants were four to six months old	Laboratory	1	12
Macaca mulatta	Bauman et al., 2006	The expression of social dominance following neonatal lesions of the amygdala or hippocampus in rhesus monkeys (*Macaca mulatta*)	laboratory	4	24
Macaca mulatta	Cheries et al., 2006	Units of visual individuation in rhesus macaques: Objects or unbound features?	field	NA	207
Macaca mulatta	Drea, 2006	Studying primate learning in group contexts: Tests of social foraging, response to novelty, and cooperative problem solving	laboratory	2	55
Macaca mulatta	Dubuc et al., 2012	Social tolerance in a despotic primate: Co-feeding between consortship partners in rhesus macaques	field	1	58
Macaca mulatta	Flombaum & Santos, 2005	Rhesus monkeys attribute perceptions to others	field	8	67
Macaca mulatta	Flombaum et al., 2004	Dynamic object individuation in rhesus macaques: A study of the tunnel effect	field	NA	246
Macaca mulatta	Fugate et al., 2008	Recognition of rhesus macaque (*Macaca mulatta*) noisy screams: Evidence from conspecifics and human listeners	laboratory	1	12
Macaca mulatta	Gazes et al., 2013	Automated cognitive testing of monkeys in social groups yields results comparable to individual laboratory-based testing	laboratory	1	12
Macaca mulatta	Hauser & Carey, 2003	Spontaneous representations of small numbers of objects by rhesus macaques: Examinations of content and format	field	7	68
Macaca mulatta	Hauser, 2007	When males call, females listen: sex differences in responsiveness to rhesus monkey, *Macaca mulatta*, copulation calls	field	7.5	58
Macaca mulatta	Hauser, Carey & Hauser, 2000	Spontaneous number representation in semi-free-ranging rhesus monkeys	field	NA	NA
Macaca mulatta	Henkel et al., 2015	Rhesus macaques (*Macaca mulatta*) recognize group membership via olfactory cues alone	field	6	74
Macaca mulatta	Higham et al., 2011	Familiarity affects the assessment of female facial signals of fertility by free-ranging male rhesus macaques	field	NA	14
Macaca mulatta	Hughes & Santos, 2012	Rotational displacement skills in rhesus macaques (*Macaca mulatta*)	field	NA	244.5
Macaca mulatta	Ioannou et al., 2015	No strings attached: physiological monitoring of rhesus monkeys *(Macaca mulatta)* with thermal imaging	sanctuary	1	5
Macaca mulatta	Johnson, 2000	Food-neophobia in semi-free ranging rhesus macaques: Effects of food limitation and food source	laboratory	1	278
Macaca mulatta	Marticorena et al., 2011	Monkeys represent others' knowledge but not their beliefs	field	NA	104.5
Macaca mulatta	Martin & Santos, 2014	The origins of belief representation: Monkeys fail to automatically represent others' beliefs	field	NA	335
Macaca mulatta	Munakata et al., 2001	Visual representation in the wild: how rhesus monkeys parse objects	field	NA	49

Table S1. The publications that tested primate cognition in a social setting published between 2000 and 2015 (inclusive), with one entry per unique combination of environment type and species per publication.

Species	Reference	Title of Article	Environment Type	Number of Groups Tested	Number of Individuals Tested
Macaca mulatta	Pfefferle et al., 2013	Female rhesus macaques discriminate unfamiliar paternal sisters in playback experiments: support for acoustic phenotype matching	field	6	72
Macaca mulatta	Pfefferle et al., 2014	Monkeys spontaneously discriminate their unfamiliar paternal kin under natural conditions using facial cues	field	4	88
Macaca mulatta	Pfefferle et al., 2015	Male rhesus macaques use vocalizations to distinguish female maternal, but not paternal, kin from non-kin	field	6	52
Macaca mulatta	Phillips & Santos, 2007	Evidence for kind representations in the absence of language: Experiments with rhesus monkeys (*Macaca mulatta*)	field	NA	126
Macaca mulatta	Phillips, Shankar & Santos, 2010	Essentialism in the absence of language? Evidence from rhesus monkeys (*Macaca mulatta*)	field	NA	251.5
Macaca mulatta	Santos et al., 2002	Object individuation using property/kind information in rhesus macaques (*Macaca mulatta*)	field	10	78
Macaca mulatta	Santos, Hauser & Spelke, 2001	Recognition and categorization of biologically significant objects by rhesus monkeys (*Macaca mulatta*): The domain of food	field	9	22.5
Macaca mulatta	Santos, Nissen & Ferrugia, 2006	Rhesus monkeys (*Macaca mulatta*) know what others can and cannot hear	field	NA	90
Macaca mulatta	Wood et al., 2008	Free-ranging rhesus monkeys spontaneously individuate and enumerate small numbers of non-solid portions	field	6	220
Macaca nigra	Micheletta & Waller, 2012	Friendship affects gaze following in a tolerant species of macaque, *Macaca nigra*	zoo	1	7
Macaca nigra	Micheletta et al., 2012	Social bonds affect anti-predator behaviour in a tolerant species of macaque, *Macaca nigra*	field	2	10
Macaca nigra	Micheletta et al., 2015	Facial expression recognition in crested macaques (*Macaca nigra*)	zoo	1	3
Macaca nigra	Micheletta et al., 2015	Familiar and unfamiliar face recognition in crested macaques (*Macaca nigra*)	zoo	1	3
Macaca nigra	Neumann et al., 2013	Personality of wild male crested macaques (*Macaca nigra*)	field	2	37
Macaca radiata	Coss et al., 2007	Threat-related acoustical differences in alarm calls by wild bonnet macaques (*Macaca radiata*) elicited by python and leopard models	field	3	NA
Macaca radiata	Coss et al., 2007	Threat-related acoustical differences in alarm calls by wild bonnet macaques (*Macaca radiata*) elicited by python and leopard models	field	1	NA
Macaca radiata	Coss et al., 2007	Threat-related acoustical differences in alarm calls by wild bonnet macaques (*Macaca radiata*) elicited by python and leopard models	field	4	NA
Macaca radiata	Coss et al., 2007	Threat-related acoustical differences in alarm calls by wild bonnet macaques (*Macaca radiata*) elicited by python and leopard models	field	2	8.5
Macaca radiata	Mangalam & Singh, 2013	Flexibility in food extraction techniques in urban free-ranging bonnet macaques, *Macaca radiata*	field	2	32
Macaca radiata	Ramakrishnan & Coss, 2000	Age differences in the responses to adult and juvenile alarm calls by bonnet macaques (*Macaca radiata*)	field	4	NA
Macaca radiata	Ramakrishnan & Coss, 2000	Age differences in the responses to adult and juvenile alarm calls by bonnet macaques (*Macaca radiata*)	field	4	NA
Macaca radiata	Ramakrishnan & Coss, 2000	Age differences in the responses to adult and juvenile alarm calls by bonnet macaques (*Macaca radiata*)	field	3	NA

Table S1. The publications that tested primate cognition in a social setting published between 2000 and 2015 (inclusive), with one entry per unique combination of environment type and species per publication.

Species	Reference	Title of Article	Environment Type	Number of Groups Tested	Number of Individuals Tested
Macaca radiata	Ramakrishnan et al., 2005	Snake species discrimination by wild bonnet macaques (*Macaca radiata*)	field	3	NA
Macaca radiata	Ramakrishnan et al., 2005	Snake species discrimination by wild bonnet macaques (*Macaca radiata*)	field	4	NA
Macaca radiata	Ramakrishnan et al., 2005	Snake species discrimination by wild bonnet macaques (*Macaca radiata*)	field	1	NA
Macaca silenus	Burkart et al., 2014	The evolutionary origin of human hyper-cooperation	zoo	1	6
Macaca silenus	Burkart et al., 2014	The evolutionary origin of human hyper-cooperation	zoo	1	6
Macaca sylvanus	Fischer & Hammerschmidt, 2001	Functional referents and acoustic similarity revisited: the case of barbary macaque alarm calls	zoo	3	48-83
Macaca sylvanus	Gustison et al., 2012	An experimental study of behavioural coping strategies in free-ranging female Barbary macaques (*Macaca sylvanus*)	zoo	1	12
Macaca sylvanus	Pfefferle et al., 2008	Male Barbary macaques eavesdrop on mating outcome: a playback study	zoo	3	18
Macaca sylvanus	Schell et al., 2011	Adult but not juvenile Barbary macaques spontaneously recognize group members from pictures	zoo	3	70
Macaca sylvanus	Teufel et al., 2007	Lack of orienting asymmetries in Barbary macaques: implications for studies of lateralized auditory processing	zoo	3	55
Mandrillus sphinx	Levréro et al., 2015	Social shaping of voices does not impair phenotype matching of kinship in mandrills	laboratory	3	13
Pan paniscus	Bardo et al., 2015	Do bimanual coordination, tool use, and body posture contribute equally to hand preferences in bonobos?	zoo	1	9
Pan paniscus	Boose, White & Meinelt, 2013	Sex differences in tool use acquisition in bonobos (*Pan paniscus*)	zoo	1	16
Pan paniscus	Clay & Zuberbühler, 2011	Bonobos extract meaning from call sequences	zoo	1	4
Pan paniscus	Cronin et al., 2015	Bonobos show limited social tolerance in a group setting: a comparison with chimpanzees and a test of the relational model	zoo	1	9
Pan paniscus	Pitman & Shumaker, 2009	Does early care affect joint attention in great apes (*Pan troglodytes, Pan paniscus, Pongo abelii, Pongo pygmaeus, Gorilla gorilla*)?	unknown	NA	3
Pan troglodytes	Bard et al., 2006	Self-awareness in human and chimpanzee infants: What is measured and what is meant by the mark and mirror test?	laboratory	NA	9
Pan troglodytes	Bonnie et al., 2007	Spread of arbitrary conventions among chimpanzees: A controlled experiment	laboratory	2	17
Pan troglodytes	Bonnie et al., 2012	Flexibility and persistence of chimpanzee (*Pan troglodytes*) foraging behavior in a captive environment	zoo	1	7
Pan troglodytes	Burkart et al., 2014	The evolutionary origin of human hyper-cooperation	zoo	1	29
Pan troglodytes	Burkart et al., 2014	The evolutionary origin of human hyper-cooperation	zoo	1	29
Pan troglodytes	Calcutt et al., 2014	Captive chimpanzees share diminishing resources	zoo	1	7

Table S1. The publications that tested primate cognition in a social setting published between 2000 and 2015 (inclusive), with one entry per unique combination of environment type and species per publication.

**Species**	**Reference**	**Title of Article**	**Environment Type**	**Number of Groups Tested**	**Number of Individuals Tested**
Pan troglodytes	Clark & Smith, 2013	Effect of a cognitive challenge device containing food and non-food rewards on chimpanzee well-being	zoo	1	6
Pan troglodytes	Crockford et al., 2012	Wild chimpanzees inform ignorant group members of danger	field	1	33
Pan troglodytes	Crockford et al., 2015	An intentional vocalization draws others' attention: A playback experiment with wild chimpanzees	field	NA	12
Pan troglodytes	Cronin et al., 2014a	Population-level variability in the social climates of four chimpanzee societies	sanctuary	4	91
Pan troglodytes	Dean et al., 2012	Identification of the social and cognitive processes underlying human cumulative culture	laboratory	8	74
Pan troglodytes	Finestone et al., 2014	The interplay between individual, social, and environmental influences on chimpanzee food choices	zoo	1	6
Pan troglodytes	Herbinger et al., 2009	Vocal, gestural, and locomoter responses of wild chimpanzees to familiar and unfamiliar intruders: A playback study	field	3	84
Pan troglodytes	Hopkins et al., 2013	Are chimpanzees really so poor at understanding imperative pointing? Some new data and an alternative view of canine and ape social cognition	laboratory	NA	18
Pan troglodytes	Hopper et al., 2007	Experimental studies of traditions and underlying transmission processes in chimpanzees	laboratory	2	16
Pan troglodytes	Hopper et al., 2011	Chimpanzees' socially maintained food preferences indicate both conservatism and conformity	laboratory	2	12
Pan troglodytes	Hopper et al., 2015	The importance of witnessed agency in chimpanzee social learning of tool use	laboratory	4	19
Pan troglodytes	Hopper et al., 2015	Captive chimpanzee foraging in a social setting: a test of problem solving, flexibility, and spatial discounting	zoo	1	6
Pan troglodytes	Hopper et al., 2015	Chimpanzees create and modify probe tools functionally: A study with zoo-housed chimpanzees	zoo	1	7
Pan troglodytes	Horner et al., 2010	Prestige affects cultural learning in chimpanzees	laboratory	2	14
Pan troglodytes	hou et al., 2007	Now you see me, now you don't: evidence that chimpanzees understand the role of the eyes in attention	laboratory	NA	116
Pan troglodytes	House et al., 2014	Task design influences prosociality in captive chimpanzees (*Pan troglodytes*)	laboratory	5	18
Pan troglodytes	Hrubesch, Preuschoft & van Schaik, 2009	Skill mastery inhibits adoption of observed alternative solutions among chimpanzees (*Pan troglodytes*)	sanctuary	2	13
Pan troglodytes	Huffman & Hirata, 2004	An experimental study of leaf swallowing in captive chimpanzees: insights into the origin of a self-medicative behavior and the role of social learning	laboratory	1	6
Pan troglodytes	Huffman et al., 2010	Leaf swallowing behavior in chimpanzees (*Pan troglodytes*): biased learning and the emergence of group level cultural differences	zoo	2	11
Pan troglodytes	Kendal et al., 2015	Chimpanzees copy dominant and knowledgeable individuals: implications for cultural diversity	laboratory	4	42
Pan troglodytes	Kutsukake et al., 2012	Individual variation in behavioural reactions to unfamiliar conspecific vocalisation and hormonal underpinnings in male chimpanzees	sanctuary	2	14
Pan troglodytes	Leavens et al., 2010	Multimodal communication by captive chimpanzees (*Pan troglodytes*)	laboratory	NA	55

Table S1. The publications that tested primate cognition in a social setting published between 2000 and 2015 (inclusive), with one entry per unique combination of environment type and species per publication.

**Species**	**Reference**	**Title of Article**	**Environment Type**	**Number of Groups Tested**	**Number of Individuals Tested**
Pan troglodytes	Lonsdorf et al., 2009	An experimental, comparative investigation of tool use in chimpanzees and gorillas	zoo	1	7
Pan troglodytes	Massen et al., 2012	Male yawning is more contagious than female yawning among chimpanzees (*Pan troglodytes*)	zoo	1	15
Pan troglodytes	Massen et al., 2013	A behavioral view on chimpanzee personality: exploration tendency, persistence, boldness, and tool-orientation measured with group experiments	zoo	1	16
Pan troglodytes	Massen et al., 2013	A behavioral view on chimpanzee personality: exploration tendency, persistence, boldness, and tool-orientation measured with group experiments	zoo	1	15
Pan troglodytes	Morimura & Mori, 2010	Effects of early rearing conditions on problem-solving skill in captive male chimpanzees (*Pan troglodytes*)	sanctuary	2	13
Pan troglodytes	Parron et al., 2008	Behavioural responses to photographs by pictorially naïve baboons (*Papio anubis*), gorillas (*Gorilla gorilla*) and chimpanzees (*Pan troglodytes)*	zoo	NA	3.5
Pan troglodytes	Pitman & Shumaker, 2009	Does early care affect joint attention in great apes (*Pan troglodytes, Pan paniscus, Pongo abelii, Pongo pygmaeus, Gorilla gorilla*)?	unknown	NA	7
Pan troglodytes	Rawlings, Davila-Ross & Boysen, 2014	Semi-wild chimpanzees open hard-shelled fruits differently across communities	sanctuary	3	56
Pan troglodytes	Russell et al., 2005	Chimpanzee (*Pan troglodytes*) intentional communication is not contingent upon food	laboratory	NA	46
Pan troglodytes	Russell et al., 2005	Chimpanzee (*Pan troglodytes*) intentional communication is not contingent upon food	laboratory	NA	13
Pan troglodytes	Schel et al., 2013	Chimpanzee alarm call production meets key criteria for intentionality	field	1	7
Pan troglodytes	Schel et al., 2013	Chimpanzee food calls are directed at specific individuals	field	1	69
Pan troglodytes	Schneider, Melis & Tomasello, 2012	How chimpanzees solve collective action problems	zoo	2	12
Pan troglodytes	Slocombe & Zuberbühler, 2005	Functionally referential communication in a chimpanzee	zoo	1	1
Pan troglodytes	Slocombe, Townsend & Zuberbühler, 2009	Wild chimpanzees (*Pan troglodytes schweinfurthii*) distinguish between different scream types: evidence from a playback study	field	1	6
Pan troglodytes	Suchak et al., 2014	Ape duos and trios: spontaneous cooperation with free partner choice in chimpanzees	laboratory	1	11
Pan troglodytes	Ushitani, Imura & Tomonaga, 2010	Object-based attention in chimpanzees (*Pan troglodytes*)	laboratory	1	2
Pan troglodytes	van Leeuwen et al., 2013	Chimpanzees (*Pan troglodytes*) flexibly adjust their behaviour in order to maximize payoffs, not to conform to majorities	zoo	1	16
Pan troglodytes	van Leeuwen et al., 2013	Chimpanzees (*Pan troglodytes*) flexibly adjust their behaviour in order to maximize payoffs, not to conform to majorities	sanctuary	1	12
Pan troglodytes	Whiten et al., 2007	Transmission of multiple traditions within and between chimpanzee groups	laboratory	6	56
Pan troglodytes	Whiten, Horner & De Waal, 2005	Conformity to cultural norms of tool use in chimpanzees	laboratory	3	39

Table S1. The publications that tested primate cognition in a social setting published between 2000 and 2015 (inclusive), with one entry per unique combination of environment type and species per publication.

**Species**	**Reference**	**Title of Article**	**Environment Type**	**Number of Groups Tested**	**Number of Individuals Tested**
Pan troglodytes	Wilson, Hauser & Wrangham, 2001	Does participation in intergroup conflict depend on numerical assessment, range location, or rank for wild chimpanzees?	field	28	50
Pan troglodytes	Wittig et al., 2014	Triadic social interactions operate across time: a field experiment with wild chimpanzees	field	1	16
Papio anubis	Laidre, 2008	Spontaneous performance of wild baboons on three novel food-access puzzles	field	1	25
Papio anubis	Meunier, Prieur & Vauclair, 2013	Olive baboons communicate intentionally by pointing	laboratory	9	9
Papio anubis	Parron et al., 2008	Behavioural responses to photographs by pictorially naïve baboons (*Papio anubis*), gorillas (*Gorilla gorilla*) and chimpanzees (*Pan troglodytes)*	laboratory	NA	27.5
Papio cynocephalus	Fischer, Cheney & Seyfarth, 2000	Development of infant baboons' responses to grade bark variants	field	1	17
Papio cynocephalus	Kitchen et al., 2003	Female baboons' responses to male loud calls	field	1	12
Papio hamadryas	Crockford et al., 2007	Baboons eavesdrop to deduce mating opportunities	field	1	9
Papio hamadryas	Kitchen et al., 2005	Male chacma baboons (*Papio hamadryas ursinus*) discriminate loud call contests between rivals of different relative ranks	field	1	10
Papio hamadryas	Kitchen et al., 2013	Male baboon responses to experimental manipulations of loud "wahoo calls": testing an honest signal of fighting ability	field	1	8
Papio hamadryas	Lemasson, Palombit, & Jubin, 2008	Friendship between males and lactating females in a free-ranging group of olive baboons (*Papio hamadryas anubis*): evidence from playback experiments	field	1	105
Papio hamadryas	Pearson et al., 2015	Crowding increases salivary cortisol but not self-directed behavior in captive baboons	laboratory	1	19
Papio hamadryas	Wittig et al., 2007	Kin-mediated reconciliation substitutes for direct reconciliation in female baboons	field	1	13
Papio hamadryas	Wittig et al., 2007	Vocal alliances in chacma baboons (*Papio hamadryas ursinus*)	field	1	13
Papio papio	Barbet & Fagot, 2011	Processing of contour closure by baboons (*Papio papio*)	laboratory	4	19
Papio papio	Bonté, Flemming & Fagot, 2011	Executive control of perceptual features and abstract relations by baboons (*Papio papio*)	laboratory	1	24
Papio papio	Claidière et al., 2014	Cultural evolution of systematically structured behaviour in a non-human primate	laboratory	1	15
Papio papio	Claidière et al., 2015	Using Automated Learning Devices for Monkeys (ALDM) to study social networks	laboratory	1	22
Papio papio	Fagot & Bonté, 2010	Automated testing of cognitive performance in monkeys: Use of a battery of computerized test systems by a troop of semi-free-ranging baboons (*Papio papio*)	laboratory	1	20
Papio papio	Fagot & De Lillo, 2011	A comparative study of working memory: Immediate serial spatial recall in baboons (*Papio papio*) and humans	laboratory	1	2
Papio papio	Fagot & Maugard, 2013	Analogical reasoning in baboons (*Papio papio*): flexible reendcoding of the source relation depending on the target relation	laboratory	1	4

Table S1. The publications that tested primate cognition in a social setting published between 2000 and 2015 (inclusive), with one entry per unique combination of environment type and species per publication.

**Species**	**Reference**	**Title of Article**	**Environment Type**	**Number of Groups Tested**	**Number of Individuals Tested**
Papio papio	Fagot & Paleressompoulle, 2009	Automatic testing of cognitive performance in baboons maintained in social groups	laboratory	3	9
Papio papio	Fagot & Parron, 2010	Relational matching in baboons (*Papio papio*) with reduced grouping requirements	laboratory	3	6
Papio papio	Fagot et al., 2013	Age-Dependant Behavioral Strategies in a Visual Search Task in Baboons (*Papio papio*) and Their Relation to Inhibitory Control	laboratory	1	18
Papio papio	Fagot et al., 2014	Effects of freely accessible computerized test systems on the spontaneous behaviors and stress level of Guinea baboons (*Papio papio*)	laboratory	2	9
Papio papio	Flemming, Thompson & Fagot, 2013	Baboons, like humans, solve analogy by categorical abstraction of relations	laboratory	1	8
Papio papio	Goujon & Fagot, 2013	Learning of spatial statistics in nonhuman primates: contextual cueing in baboons (*Papio papio*)	laboratory	1	25
Papio papio	Huguet et al., 2014	Cognitive control under social influence in baboons	laboratory	NA	11
Papio papio	Maciej et al., 2013	Social monitoring in a multilevel society: a playback study with male Guinea baboons	field	2	12
Papio papio	Marzouki et al., 2014	Baboons' response speed is biased by their moods	laboratory	2	6
Papio papio	Maugard, Marzouki & Fagot, 2013	Contribution of working memory processes to relational matching-to-sample performance in baboons (*Papio Papio*)	laboratory	1	10
Papio papio	Parron & Fagot, 2010	First- and second-order configural sensitivity for greeble stimuli in baboons	laboratory	1	3
Papio papio	Pope et al., 2015	Baboons (*Papio papio*), but not humans, break cognitive set in a visuomotor task	laboratory	1	15
Papio ursinus	Bergman & Kitchen, 2009	Comparing responses to novel objects in wild baboons (*Papio ursinus*) and geladas (*Theropithecus gelada*)	field	1	78
Papio ursinus	Carter et al., 2012	How not to measure boldness: novel object and antipredator responses are not the same in wild baboons	field	2	57
Papio ursinus	Carter et al., 2014	Personality predicts the propensity for social learning in a wild primate	field	2	55
Papio ursinus	Noser & Byrne, 2015	Wild chacma baboons (*Papio ursinus*) remember single foraging episodes	field	1	24
Pithecia pithecia	Burkart et al., 2014	The evolutionary origin of human hyper-cooperation	zoo	1	5
Pongo abelii	Forss et al., 2015	Contrasting responses to novelty by wild and captive orangutans	field	NA	28
Pongo abelii	Forss et al., 2015	Contrasting responses to novelty by wild and captive orangutans	zoo	1	7
Pongo abelii	Pitman & Shumaker, 2009	Does early care affect joint attention in great apes (*Pan troglodytes, Pan paniscus, Pongo abelii, Pongo pygmaeus, Gorilla gorilla*)?	unknown	NA	NA
Pongo abelii	Scheumann & Call, 2006	Sumatran orangutans and a yellow-cheeked crested gibbon know what is where	zoo	1	6
Pongo pygmaeus	Forss et al., 2015	Contrasting responses to novelty by wild and captive orangutans	field	NA	28

Table S1. The publications that tested primate cognition in a social setting published between 2000 and 2015 (inclusive), with one entry per unique combination of environment type and species per publication.

**Species**	**Reference**	**Title of Article**	**Environment Type**	**Number of Groups Tested**	**Number of Individuals Tested**
Pongo pygmaeus	Lehner et al., 2011	Can captive orangutans (Pongo pygmaeus abelii) be coaxed into cumulative build-up of techniques?	zoo	1	7
Pongo pygmaeus	Pitman & Shumaker, 2009	Does early care affect joint attention in great apes (*Pan troglodytes, Pan paniscus, Pongo abelii, Pongo pygmaeus, Gorilla gorilla*)?	unknown	NA	NA
Presbytis thomasi	Wich & de Vries, 2006	Male monkeys remember which group members have given alarm calls	field	12	12
Presbytis thomasi	Wich & Sterck, 2003	Possible audience effect in Thomas langurs (primates; *Presbytis thomasi*): An experimental study on male loud calls in response to a tiger model	field	12	12
Presbytis thomasi	Wich et al., 2002	Playbacks of loud calls to wild Thomas langurs (Primates; *Presbytis thomasi*): the effect of location	field	12	NA
Presbytis thomasi	Wich et al., 2004	Thomas langurs (*Presbytis thomasi*) discriminate between calls of young solitary versus older group-living males: a factor in avoiding infanticide?	field	10	NA
Propithecus verreauxi	Fichtel & Kappeler, 2002	Anti-predator behavior of group-living Malagasy primates: mixed evidence for a referential alarm call system	field	4	6
Propithecus verreauxi	Fichtel, 2005	Reciprocal recognition of sifaka (*Propithecus verrauxi verreauxi*) and redfronted lemur (*Eulemur fulvus rufus*) alarm calls	field	4	8
Propithecus verreauxi	Fichtel, 2008	Ontogeny of conspecific and heterospecific alarm call recognition in wild verreaux's sifakas (*Propithecus verreauxi verreauxi*)	field	12	19
Rhinopithecus roxellana	Fu et al., 2013	Free-ranging Sichuan snub-nosed monkeys *Rhinopithecus roxellana*: Neophobia, neophilia, or both?	field	1	24
Saguinus fuscicollis	Bicca-Marques & Garber, 2004	Use of spatial, visual, and olfactory information during foraging in wild nocturnal and diurnal anthropoids: A field experiment comparing *Aotus, Callicebus*, and *Saguinu*s	field	2	9
Saguinus fuscicollis	Bradley & McClung, 2015	Vocal divergence and discrimination of long calls in tamarins: a comparison of allopatric populations of *Saguinus fuscicollis nigrifrons* and *S. f. lagonotus*	field	5	NA
Saguinus fuscicollis	Hardie & Buchanan-Smith, 2000	Responses of captive single- and mixed-species groups of *Saguinus* to novel nonthreatening objects	zoo	7	25
Saguinus fuscicollis & Saguinas mystax	Kirchhof & Hammerschmidt, 2006	Functionally referential alarm calls in tamarins (*Saguinus fuscicollis and S. mystax*) - evidence from playback experiments	field	3	21
Saguinus imperator	Bicca-Marques & Garber, 2004	Use of spatial, visual, and olfactory information during foraging in wild nocturnal and diurnal anthropoids: A field experiment comparing *Aotus, Callicebus*, and *Saguinus*	field	2	10
Saguinus imperator	Day et al., 2003	Neophilia, innovation and social learning: A study of intergeneric differences in callitrichid monkeys	zoo	3	9
Saguinus labiatus	Hardie & Buchanan-Smith, 2000	Responses of captive single- and mixed-species groups of *Saguinus* to novel nonthreatening objects	zoo	6	21
Saguinus oedipus	Burkart et al., 2014	The evolutionary origin of human hyper-cooperation	laboratory	1	5
Saguinus oedipus	Campbell & Snowdon, 2009	Can auditory playback condition predator mobbing in captive-reared *Saguinus oedipus*?	laboratory	1	6
Saguinus oedipus	Day et al., 2003	Neophilia, innovation and social learning: A study of intergeneric differences in callitrichid monkeys	zoo	1	4

Table S1. The publications that tested primate cognition in a social setting published between 2000 and 2015 (inclusive), with one entry per unique combination of environment type and species per publication.

**Species**	**Reference**	**Title of Article**	**Environment Type**	**Number of Groups Tested**	**Number of Individuals Tested**
Saguinus oedipus	Matthews & Snowdon, 2011	Long-term memory for calls of relatives in cotton-top tamarins (*Saguinus oedipus*)	laboratory	NA	22
Saguinus oedipus	Roush & Snowdon, 2000	Quality, quantity, distribution and audience effects on food calling in cotton-top tamarins	laboratory	7	NA
Saguinus oedipus	Snowdon & Boe, 2003	Social communication about unpalatable foods in tamarins (*Saguinus oedipus*)	laboratory	8	44
Saguinus oedipus	Zahed et al., 2008	Male parenting and response to infant stimuli in the common marmoset (*Callithrix jacchus*)	laboratory	15	15
Saimiri boliviensis	Hopper et al., 2013	Dissecting the mechanisms of squirrel monkey (*Saimiri boliviensis*) social learning	laboratory	3	34
Saimiri sciureus	Burkart et al., 2014	The evolutionary origin of human hyper-cooperation	zoo	1	19
Saimiri sciureus	Claidière et al., 2013	Diffusion dynamics of socially learned foraging techniques in squirrel monkeys	zoo	2	27
Saimiri sciureus	McCowan & Newman, 2000	The role of learning in chuck call recognition by squirrel monkeys (*Saimiri sciureus*)	laboratory	3	17
Saimiri sciureus	Soltis et al., 2002	Squirrel monkey chuck call: vocal response to playback chucks on acoustic structure and affiliative relationship with caller	laboratory	2	10
Sapajus libidinosus	Fragaszy et al., 2013	Wild bearded capuchin monkeys (*Sapajus libidinosus*) strategically place nuts in a stable position during nut-cracking	field	1	10
Sapajus libidinosus	Howard et al., 2015	Landscape influences on the natural and artificially manipulated movements of bearded capuchin monkeys	field	1	8
Simias concolor	Yorzinski & Ziegler, 2007	Do naïve primates recognize the vocalizations of felid predators?	field	NA	88
Theropithecus gelada	Bergman & Kitchen, 2009	Comparing responses to novel objects in wild baboons (*Papio ursinus*) and geladas (*Theropithecus gelada*)	field	3	79
Theropithecus gelada	Bergman, 2010	Experimental evidence for limited vocal recognition in a wild primate: implications for the social complexity hypothesis	field	4	18
Theropithecus gelada	le Roux & Bergman, 2012	Indirect rival assessment in a social primate, *Theropithecus gelada*	field	NA	35
Varecia variegata	Burkart et al., 2014	The evolutionary origin of human hyper-cooperation	laboratory	1	6
Varecia variegata	Stoinski, Drayton & Price, 2011	Evidence of social learning in black-and-white ruffed lemurs (*Varecia variegata*)	zoo	1	8
